# Adapting a Telephone-Based, Dyadic Self-management Program to Be Delivered Over the Web: Methodology and Usability Testing

**DOI:** 10.2196/43903

**Published:** 2023-06-16

**Authors:** Ranak Trivedi, Sierra Kawena Hirayama, Rashmi Risbud, Madhuvanthi Suresh, Marika Blair Humber, Kevin Butler, Alex Razze, Christine Timko, Karin Nelson, Donna M Zulman, Steven M Asch, Keith Humphreys, John D Piette

**Affiliations:** 1 Center for Innovation to Implementation Veteran Affairs Palo Alto Health Care System Menlo Park, CA United States; 2 Stanford University Palo Alto, CA United States; 3 Learning Systems International Metcor Washington, DC United States; 4 Veteran Affairs Puget Sound Health Care System Seattle, WA United States; 5 Veteran Affairs Ann Arbor Health Care System Ann Arbor, MI United States

**Keywords:** dyadic, eHealth, behavioral interventions, self-management, caregiver, web-based, interventions, stress management, self-care

## Abstract

**Background:**

The COVID-19 pandemic has amplified the need for web-based behavioral interventions to support individuals who are diagnosed with chronic conditions and their informal caregivers. However, most interventions focus on patient outcomes. Dyadic technology–enabled interventions that simultaneously improve outcomes for patients and caregivers are needed.

**Objective:**

This study aimed to describe the methodology used to adapt a telephone-based, facilitated, and dyadic self-management program called Self-care Using Collaborative Coping Enhancement in Diseases (SUCCEED) into a self-guided, web-based version (web-SUCCEED) and to conduct usability testing for web-SUCCEED.

**Methods:**

We developed web-SUCCEED in 6 steps: ideation—determine the intervention content areas; prototyping—develop the wireframes, illustrating the look and feel of the website; prototype refinement via feedback from focus groups; finalizing the module content; programming web-SUCCEED; and usability testing. A diverse team of stakeholders including content experts, web designers, patients, and caregivers provided input at various stages of development. Costs, including full-time equivalent employee, were summarized.

**Results:**

At the ideation stage, we determined the content of web-SUCCEED based on feedback from the program’s original pilot study. At the prototyping stage, the principal investigator and web designers iteratively developed prototypes that included inclusive design elements (eg, large font size). Feedback about these prototypes was elicited through 2 focus groups of veterans with chronic conditions (n=13). Rapid thematic analysis identified two themes: (1) web-based interventions can be useful for many but should include ways to connect with other users and (2) prototypes were sufficient to elicit feedback about the esthetics, but a live website allowing for continual feedback and updating would be better. Focus group feedback was incorporated into building a functional website. In parallel, the content experts worked in small groups to adapt SUCCEED’s content, so that it could be delivered in a didactic, self-guided format. Usability testing was completed by veterans (8/16, 50%) and caregivers (8/16, 50%). Veterans and caregivers gave web-SUCCEED high usability scores, noting that it was easy to understand, easy to use, and not overly burdensome. Notable negative feedback included “slightly agreeing” that the site was confusing and awkward. All veterans (8/8, 100%) agreed that they would choose this type of program in the future to access an intervention that aims to improve their health. Developing and maintaining the software and hosting together cost approximately US $100,000, excluding salary and fringe benefits for project personnel (steps 1-3: US $25,000; steps 4-6: US $75,000).

**Conclusions:**

Adapting an existing, facilitated self-management support program for delivery via the web is feasible, and such programs can remotely deliver content. Input from a multidisciplinary team of experts and stakeholders can ensure the program’s success. Those interested in adapting programs should have a realistic estimate of the budget and staffing requirements.

## Introduction

### Background

Evidence-based behavioral interventions provide individuals with chronic health conditions with a variety of tools for making behavior changes, monitoring their health status, and communicating effectively with health care teams. These behaviors are collectively termed as “self-management,” and those with chronic conditions are often supported by a relative or friend in maintaining these behaviors. In this way, the stress of managing chronic conditions is shared by the person with the diagnosis and their relatives and friends who provide direct care, self-management support, encouragement, and emotional support [1-3].

Dyadic behavioral interventions are designed to support both the patient and their caregivers in coping with emotional and practical challenges [4]. Well-designed, dyadic programs can improve patients’ adherence to self-management recommendations, quality of life, and self-efficacy while reducing hospitalization rates [5,6]. Most [7] dyadic interventions require real-time communication between intervention recipients and health coaches or facilitators, either in person or via telephone. Asynchronous communication [4,8,9] between program participants and facilitators is less common. Technology-enabled interventions have the potential to decrease the amount of resources needed per patient-caregiver dyad, and as a consequence, such programs can be more scalable and more easily modified and personalized [10].

Distance technology is particularly important for self-management interventions because the goal of such interventions is to change users’ behavior in their day-to-day lives. Technology-enabled dyadic self-management interventions have been found to be effective for individuals with many clinical conditions, notably heart failure, diabetes, cancer, and depression [11-13]. Systematic reviews have shown that technology-based interventions can improve knowledge, behaviors, and clinical outcomes for patients and their informal caregivers [14-16]. However, key gaps remain, as summarized by a systematic review of 101 studies representing 52 unique dyadic eHealth interventions [17]. First, only 18 interventions focused on adult dyads, and 9 focused specifically on adult dyads managing cancer. This highlights the research gap in technology-enabled interventions that address the needs of adult dyads managing common chronic conditions. Second, dyadic interventions developed so far have been focused on outcomes of the care recipient, with only 1 in 5 studies including outcomes for the caregiver. Third, dyadic interventions rarely address the strain on the dyadic relationship caused by chronic illness management, even though such challenges have been well documented [18-22]. We sought to develop a dyadic technology–enabled intervention that would simultaneously support the needs of patients and caregivers and their interpersonal relationship.

We previously developed and successfully pilot-tested a telephone-based dyadic self-management program called Self-care Using Collaborative Coping Enhancement in Diseases (SUCCEED) [23]. Over six 1-hour sessions, patient-caregiver dyads learned and practiced cognitive behavioral skills to reduce individual and relationship stress, improve positive emotions, improve communication and collaboration, increase pleasant activities, and maintain behavior changes despite challenges. Weekly homework involved developing an action plan to practice and sustain new skills. A pilot test of SUCCEED showed high acceptability and feasibility. However, as has been documented by others [24], dyads found it logistically challenging to find time weekly to participate in synchronous sessions with the facilitator. In addition, as sessions required a live facilitator, programs such as SUCCEED often have long wait times and limited reach [25].

### Objective

We developed a web-based, self-guided version of SUCCEED to address barriers to accessing the program and challenges associated with scaling up. In this paper, we defined a process of adapting existing dyadic behavioral interventions to web-based delivery platforms and estimated the resources necessary to complete this rigorously. Specifically, we described the process of adapting SUCCEED for web-based use and reported results of initial usability testing. We also reported estimates of the costs and staffing resources required for this adaptation to support future planning.

## Methods

### Ethics Approval

This study was reviewed and approved by the institutional review board at the Department of Veterans Affairs (VA) Palo Alto Health Care System and Stanford University (institutional review board protocol #40022) and received approval from the local scientific review committee and the health system’s information security officer (#TRI0008). All study participants provided verbal informed consent, as a waiver of documentation had been approved.

### Overview of the Study

The analyses presented in this paper represent the primary purpose of the study. Study data were deidentified before the analysis. A code linking the study ID numbers with the identifying information was maintained by the study team. All data including linked data files were stored behind a firewall on secured drives. Study participants in the focus groups were compensated with US $25, and those who participated in the usability testing were compensated with US $50. This study was conducted between 2016 and 2020. The web design was completed between 2016 and 2019 (before the COVID-19 pandemic), and usability testing was conducted between 2019 and 2021 (overlapping with the COVID-19 pandemic).

**Figure 1:**
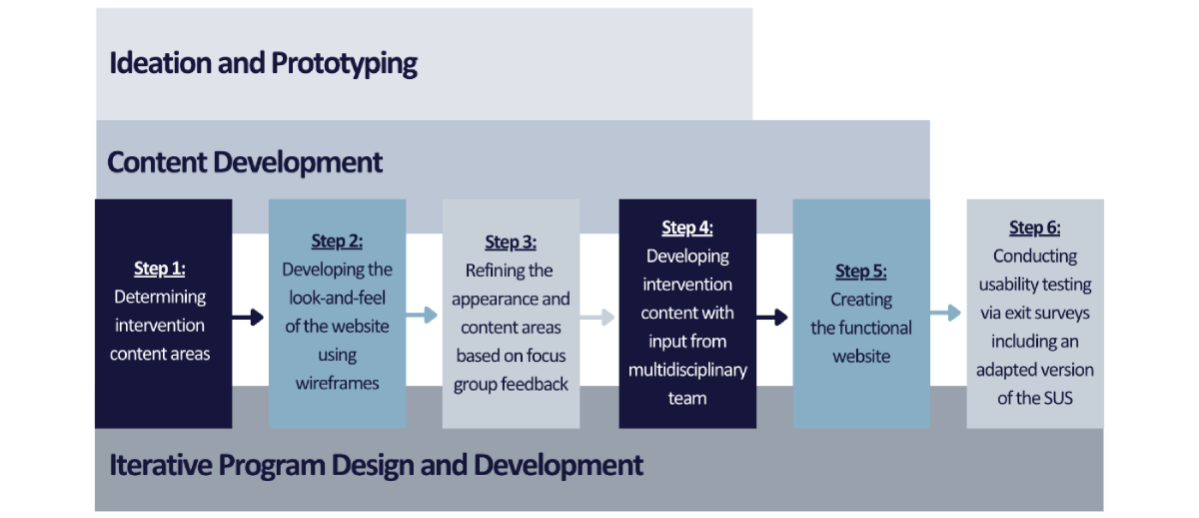
Timeline and development process for adapting the Self-care Using Collaborative Coping Enhancement in Diseases intervention to a web-based format. SUS: System Usability Scale.

Our study was informed by input from multiple stakeholders. We established and sustained partnerships with the Veteran and Family Advisory Board at our institution and obtained their input at each stage of the study. We assembled a multidisciplinary team of content experts, methodologists, administrative personnel, web designers, and software engineers. Stakeholders’ input guided all aspects of the program development process, which included ideation, prototyping, refining the prototype, finalizing the module content and scripts, programming web-SUCCEED, and usability testing (Figure 1) [26]. We strived to use human-centered design principles in the development process, including input from patients and their caregivers at each stage, to ensure that our eventual program was not only theoretically sound but also valuable to end users. In this study, we were guided by the User Experience Honeycomb [27], a user experience framework, which notes that for a product to be valuable, it should be useful, usable, desirable, findable, credible, and accessible. In addition, to ensure human-centeredness, we optimized the user experience by eliciting users’ needs and values, including making our technology accessible across abilities and disabilities.

### Step 1—Ideation

The usefulness and credibility of the program were established during the development of the SUCCEED intervention [23]. The content foci for SUCCEED were based on cognitive behavioral theory and dyadic coping and communication frameworks, including the Dyadic Behavioral Health Change Model [23]. Training objectives were drawn from the original SUCCEED intervention and included skills to manage stress and negative emotions, improve interpersonal communication and collaboration, and build a fulfilling life in the context of chronic health conditions. Disease-specific content was eliminated from session 1 based on feedback we received from participants in the SUCCEED pilot study. The technology platform was selected based on input from members of the local Veteran and Family Council, who shared that veterans preferred web-based programs over mobile apps, primarily because the content was easy to view on a large screen.

### Step 2—Prototyping

The web design was completed by a company experienced in developing learning systems for the Veterans Health Administration and was intended to maximize the desirability, usability, and accessibility domains of SUCCEED. We were committed to the principles of equity and inclusivity in our web-based platform and made accessibility central to the design. Our platform was designed to be compliant with Section 508 of the Rehabilitation Act [28]. Amended in 1998, the Rehabilitation Act is a federal law requiring federal agencies to “provide individuals with disabilities equal access to electronic information and data comparable with those who do not have disabilities, unless an undue burden would be imposed on the agency.” Section 508 documents the technical standards that must be met to ensure that technology platforms are accessible for individuals with physical, sensory, or cognitive disabilities. In collaboration with the web designers, the project team developed initial wireframes, that is, nonfunctional schematics depicting the framework and flow of the website. We incorporated inclusive design elements in the prototype, including using a sans serif font, large font size, and minimalist design, which are recommended for those with visual disability. After the wireframes were established, we added graphics, color, and styling. We designed the home page to include an introductory video and the content area of SUCCEED. Initial design concepts for module 1 were carried through in the planning of all 3 skills training modules, each of which included an overview of the content, a link to the action plan review, links to the module content, and a link to a blank action plan to be completed as homework.

### Step 3—Refining the Prototype

#### Overview

We assessed the desirability of the program by obtaining feedback from focus group participants about the website prototypes created in Microsoft PowerPoint (Microsoft Corporation). Focus group participants were recruited via flyers posted in VA clinics that included a phone number to contact the study team if they were interested. Study participants were required to be aged at least 18 years and either have at least one chronic condition (veteran) or be a caregiver of someone with at least one chronic condition (caregiver). Participants were provided a stipend worth US $25 for participating in a 1-hour focus group to provide feedback about web-SUCCEED. Focus groups were facilitated by an expert in qualitative research and assisted by a study team member who took notes. A structured interview guide was developed. During the focus groups, participants were provided with the rationale of the web-SUCCEED program, and feedback was elicited about the utility of the program, color scheme, layout, stock photos, and readability. Sample questions included, “Would you use an online program to manage your health?” “What do you like most about this website? What do you like the least?” and “What about the webpage resonates with you, and what does not?” Participants were provided with writing materials and encouraged to provide both verbal and written feedback. Focus groups were audio recorded and professionally transcribed. Focus group data were analyzed for themes around the usefulness of a web-based format and the aesthetics of the prototypes. Rapid analytic approaches were used to tabulate responses to the questions that would help address the questions, “Would an online program be useful in helping veterans with chronic health conditions manage their health” and “How accessible and appealing were the prototypes of web-SUCCEED?”

Overall, 2 focus groups with a total of 13 veterans were conducted. Unfortunately, no caregivers volunteered for the focus groups. Typical of VA patient samples, of the 13 participants, 12 (92%) participants across the 2 focus groups were men. Our sample was ethnically diverse (6/13, 46% were from underrepresented minority groups). Of these, 4 were African American (31%), and 2 (15%) were multiracial. Among the 13 participants, the mean age was 68.3 years, 9 (69%) were retired, 2 (15%) were disabled, and the remaining (n=2, 15%) were employed. All participants (13/13, 100%) had at minimum a high school degree and 31% (4/13) had a college degree or higher level of education. Of the 13 participants, 5 (38%) participants lived alone. Participants reported receiving care for their health conditions from a variety of caregivers, mainly significant others (3/13, 23%) and children (3/13, 23%).

Preliminary coding was conducted by the study team, and themes were finalized by the principal investigator (PI). In total, 2 themes were identified through the focus groups.

#### Theme 1—Web-Based Interventions Can Be Useful for Many but Should Include Ways to Connect With Other Users

Participants noted that web-based programs would be useful because they helped overcome logistical barriers and noted that they found MyHealtheVet to be useful in communicating with their health care team. When asked whether participants would use a program such as web-SUCCEED, participants noted, “I would try it” and “I think it’d be a good idea.” However, web-based programs were not universally acceptable, and participants expressed concerns around the security of medical information and that web-based programs would reduce interpersonal connections. A participant summarized the following:

But it sounds like everybody’s saying that if you have a chance to do it, do it both ways...there are some people, you know, it’s the actual face to face is better for certain people. And some people, like me...sometimes I just want to be on the computer.

Participants noted that web-SUCCEED should include strategies to communicate with the study team and with one another. We elicited feedback about whether discussion boards for communication among patient users should be included, as these have been shown to enhance programmatic engagement in self-management programs [29,30]. Although some participants (7/13, 54%) supported having a way to connect with other participants, other patients in the focus groups disagreed, noting that web-based connections were not as desirable as personal connections. People noted that preferences for discussion boards likely varied. A veteran said the following:

I would use it. My wife wouldn’t use it...I like the idea of being able to send little messages back.

Alternatives to discussion boards were suggested, including group meetings at set intervals where people could meet in person or via teleconference calls to share stories. A participant compared this with the focus group participation and noted the following:

This type of sit-down group [is] useful...for the interpersonal connections that we’re able to make.

Another participant noted the following:

But you can’t throw computers away. And so the computers are just adjunct.

Focus group respondents suggested that we clearly include our contact information on the website and make it easy to communicate directly with the study team. Focus groups also addressed the pros and cons of a combined discussion board for veterans and caregivers.

#### Theme 2—Prototypes Were Sufficient to Elicit Feedback About the Esthetics, but a Live Website Would Elicit Better Feedback

Focus group participants indicated that they liked the color scheme and design depicted by the prototype and that they could relate to the people in the photos. Participants noted that the prototypes were “straightforward,” “not convoluted,” and “simple, but effective.” When asked, participants preferred having the choice of navigating between modules rather than having to follow a forced order. They also preferred a program they could engage in individually, even if their caregiver did not want to participate or if they did not have a caregiver.

Feedback from focus groups was summarized using templates provided by the design team to modify the website. This information was used to inform an extramural grant proposal that was important for securing funds to develop the website for web-SUCCEED. The multidisciplinary team supported by that grant included the lead (a clinical health psychologist and caregiver expert), web designers, psychologists, internists, and an epidemiologist.

### Step 4—Finalizing the Module Content and Scripts

This step focused on the usefulness and credibility of the program while incorporating previous feedback related to other domains. We used specification documents from the web design team as an outline for developing the content, layout, flow, and script for the narration. We sought to develop the module narratives without degrading the key behavioral content from SUCCEED. The initial narrative script was developed by the research team and 4 experts (2 internists, 1 cardiologist, and 1 clinical psychologist) based on the content in the original SUCCEED modules, and the remainder of the experts provided feedback. The finalized modules were the following:

1. Introduction
2. Module 1—skills to reduce stress and improve positive emotions
3. Module 2—skills to reduce relationship stress and improve interpersonal relationships, which combined SUCCEED sessions 4 and 5
4. Module 3—building a fulfilling life and maintaining behavior change, which was SUCCEED session 6

**Figure 2:**
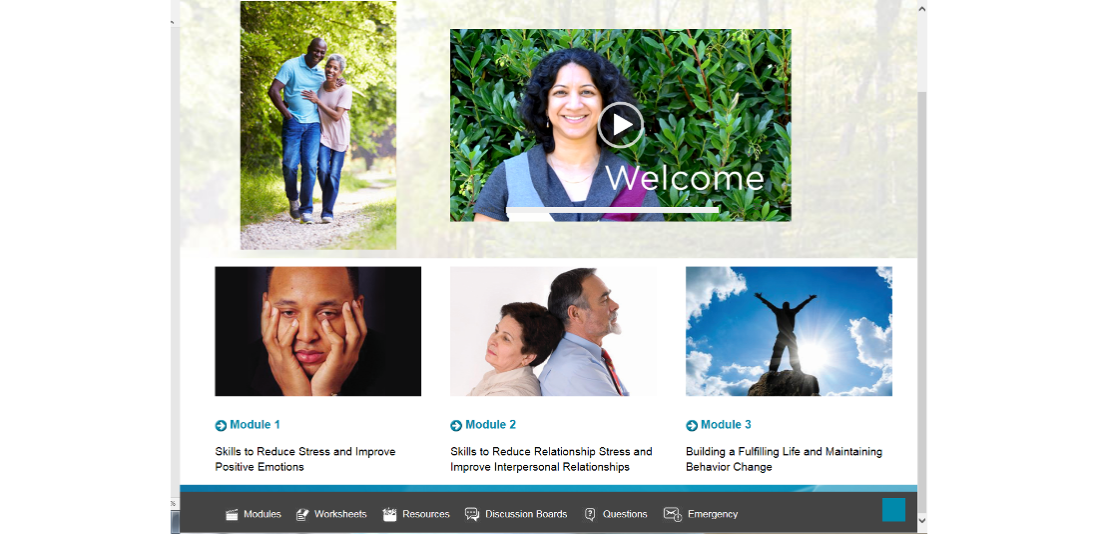
Web-based Self-care Using Collaborative Coping Enhancement in Diseases home page.

**Figure 3:**
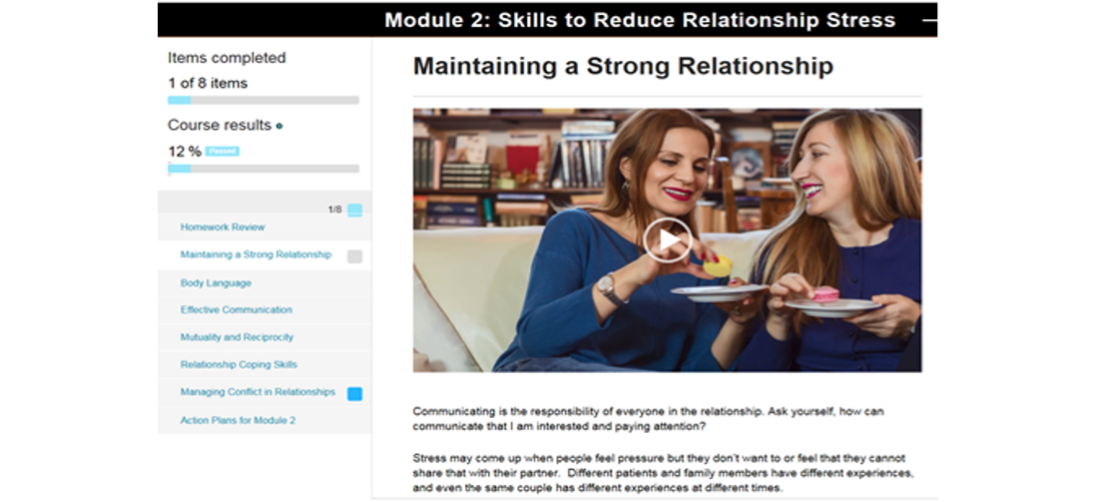
Example landing page.

Example screenshots are provided in Figures 2 and 3. The introduction module provided a welcome message, an overview of the program and its rationale, steps to develop an action plan, and tips to navigate the website. The site required that all participants complete the introduction module before proceeding to subsequent modules, which was designed based on focus group feedback to allow modules to be completed in any order at the user’s preference. At the end of each module, participants developed an action plan to practice new skills and were prompted to provide feedback regarding the relevance of the content. We designed a resource page that included skills training exercises; a list of VA resources, including links to other VA programs that support those with chronic disease and their caregivers; community resources such as the Family Caregiver Alliance; and popular chronic disease management apps. All links referenced reliable information about chronic diseases for patients and their caregivers. Worksheets and action plan homework were also available to be downloaded from the website.

### Step 5—Programming Web-SUCCEED

We chose WordPress as our platform, which is both Section 508 compliant and widely used to support behavioral interventions in the VA. In addition to the design elements that were included in the prototype, we provided voice-over narration to assist those with visual disabilities and verbatim written text of all module content for those with hearing disabilities. When accessed via a PC, the web pages did not require scrolling, which streamlined access to information and made navigating the website easy for those with motor disabilities. We developed audio recordings of key exercises that could be accessed on the resources web page. Our team reviewed 600 stock photographs to choose those that represented a diversity of skin tones, ages, and relationships (eg, old woman–young woman, 2 men and a child, and Black man–Black woman).

To allow user access while ensuring system security, each participant was required to have an email address, which served as their default user ID that could not be changed. Users could change their display names after the initial log-on. In addition to unique IDs, each user set their own password, and we were able to log session activity according to participant. Users also were given a unique study identifier.

A combined discussion board for patients and their caregivers was developed, and posts were reviewed by the moderator before being made public to the group. On the basis of the focus groups’ feedback, we configured 2 ways to communicate with the program team. A “Questions” button on the navigation bar allowed participants to connect with the team for nonurgent matters, such as with inquiries about creating their first action plan. An “Emergency” button was placed on the navigation bar, and clicking this button generated a prefilled message that had the sender’s email address and an optional text field. This page also listed the phone numbers of the National Suicide Help Line and a reminder to call 911 in case of an emergency. If patients or caregivers generated an urgent message, the study PI, a licensed clinical psychologist, would be alerted immediately via email and would reach out to evaluate the situation and respond as needed. However, this did not occur during testing. The initial version of the website did not render well on iPads; therefore, these were not recommended for use with the intervention.

### Step 6—Usability Testing

#### Recruitment and Eligibility

Veterans and caregivers were recruited in person through the VA Palo Alto Health Care System (VAPAHCS) primary care clinics, women’s health clinics, and nephrology departments and via flyers placed throughout the VAPAHCS Palo Alto and Menlo Park health system campuses. Caregiver coordinators at VAPAHCS also helped disseminate information about the study and identify caregivers.

Studies have suggested that 80% to 85% of all usability problems can be uncovered by having 4 participants navigate web-based tools [31]; however, more recent evaluations suggest increasing that number [32]. Therefore, our goal was to recruit at least 5 veterans and 5 caregivers to participate in usability testing. Veterans were eligible if they were aged ≥18 years, had been diagnosed with a chronic condition that had clear self-management recommendations, and had a relative or friend who helped them manage their health condition. We excluded veterans who had cognitive impairments; were receiving intravenous chemotherapy, radiation therapy, or hemodialysis; lived in a skilled nursing facility; or had a paid home-based caregiver (eg, home nurse aide) who provided >50% of their home care. Eligible caregivers were aged at least 18 years, had been identified as primary caregivers by the veteran, and were not being treated for cancer and not undergoing hemodialysis.

Potential patients and caregivers were further screened for challenges with self-management and their degree of comfort with technology. To screen for challenges with self-management, we used an adapted version of the Diabetes Distress Screening scale that did not use diabetes-specific language [33]. Veterans were asked to rate the extent to which they felt overwhelmed by the demands of living with chronic illnesses and were failing regimens in any of their conditions. Caregivers were asked to what extent they felt overwhelmed in managing their care recipient’s chronic conditions and were failing in managing their care recipient’s conditions. Participants rated each item on a scale of 1 (not a problem) to 6 (serious problem). Veterans and caregivers were eligible if their average rating was at least 3 or if the sum of the 2 items was ≥6. Eligible and interested veterans and caregivers provided informed consent and contact information, including email. Although our goal was to create a program that would eventually address dyadic needs, we also allowed veterans and caregivers to participate individually for this usability study if they met all other criteria but the other person was not interested in participating.

We iteratively refined the retention methods. After noting that previous participants were not progressing through the program, we sought to re-engage participants and encourage them to complete the study. These procedures involved calling 3 times at various times of day for participants who had provided consent but had not begun the program. For participants who had begun the program, we called and left voice mails multiple times, varying the times of day and calling over the weekend. One of our initial challenges was that our study team was unable to check website use for progress. Eventually, a study team member was trained by the web development team to check website use and progress. This allowed us to proactively reach out to participants who were not progressing through the program. Of the 53 individuals who were approached and were eligible, 17 (32%) patients and 16 (30%) caregivers were enrolled (total: 33/53, 62%). Our recruitment rate was higher than the average recruitment rate of 51.2% found in a systematic review of 53 trials of dyadic behavioral interventions [34]. Of these 33 participants, 8 (24%) patients and 8 (24%) caregivers completed the usability testing. The main reason for participants’ withdrawal was the stress of the pandemic.

#### Measures and Procedures

Once consented, participants were asked to complete baseline surveys to capture detailed demographic information including their age, gender, race, ethnicity, education, and marital status and the relationship between the veteran and caregiver. Participant’s financial status was measured using a tool in which respondents rated the extent to which they could afford necessities and luxuries. Baseline surveys also assessed participants’ use of and comfort with technology. We asked a series of questions that assessed the frequency with which respondents used computers in their daily lives, the ways in which they used computers (eg, spreadsheets, Word documents, and internet), and their comfort with technology (eg, “I feel very comfortable learning how to use new programs” and “I try to avoid using computers when possible.”) After completing the baseline measures, participants were emailed a unique link to log in to web-SUCCEED and were encouraged to complete all modules.

Upon the completion of the program, we emailed a Qualtrics link with follow-up surveys designed to assess the usability of web-SUCCEED. Usability was measured using a modified version of the Systems Usability Scale [35], which scored items on a 7-point scale (instead of the currently recommended 5-point scale) where 1 was labeled “Strongly Agree,” 4 was labeled “Neutral,” and 7 was labeled “Strongly Disagree.” Example items include, “I thought this program was easy to understand” and “I would need help from a technical support person to be able to use this program.” The reliability coefficient α of the Systems Usability Scale is excellent, ranging between 0.85 and 0.92, and its concurrent validity correlation coefficient is 0.81 [36,37]. Scores >4 are coded as “agree,” scores of exactly 4 are coded as “neutral,” and scores <4 were coded as “disagree.” An exit survey via Qualtrics or telephone was used to provide feedback about the program’s length, content, mode of delivery, and perceived utility and study burden. We tracked program costs through personnel full-time equivalent employee, costs of building the website, and costs to conduct usability testing.

It should be noted that we had designed a “think-aloud” protocol using the Health IT Usability Evaluation Model framework [38]. However, we were unable to conduct them. Neither veterans nor caregivers were interested in attending 4 in-person sessions to assess the usability of each module. Once COVID-19 shelter-in-place restrictions were established, we offered to conduct these interviews over telephone; however, only 6% (1/16) of the participants agreed to this modified approach. Therefore, we relied on the usability survey and exit interviews to obtain feedback.

## Results

### Overview

Overall, 16 participants including 8 (50%) veterans and 8 (50%) caregivers participated in usability testing. All participants (16/16, 100%) completed at least one module and the usability survey (Table 1). All veterans (8/8, 100%) in this study were men, whereas all caregivers (8/8, 100%) were women. Most participants (10/16, 63%) identified as White, except for a veteran who identified as having multiple ethnicities (Black, White, and Native American) and a caregiver who identified as Black or African American. Most veterans and caregivers (13/18, 72%) had at least a high school education, and most (11/16, 69%) reported that they were able to afford to pay their bills. Overall, one-third (5/16, 31%) of our participants were disabled (2/8, 25% of veterans; 3/8, 38% of caregivers); however, the type of disability was not documented. All veterans (8/8, 100%) reported that a spouse or partner cared for them, whereas all caregivers (8/8, 100%) reported caring for themselves.

**Figure 4:**
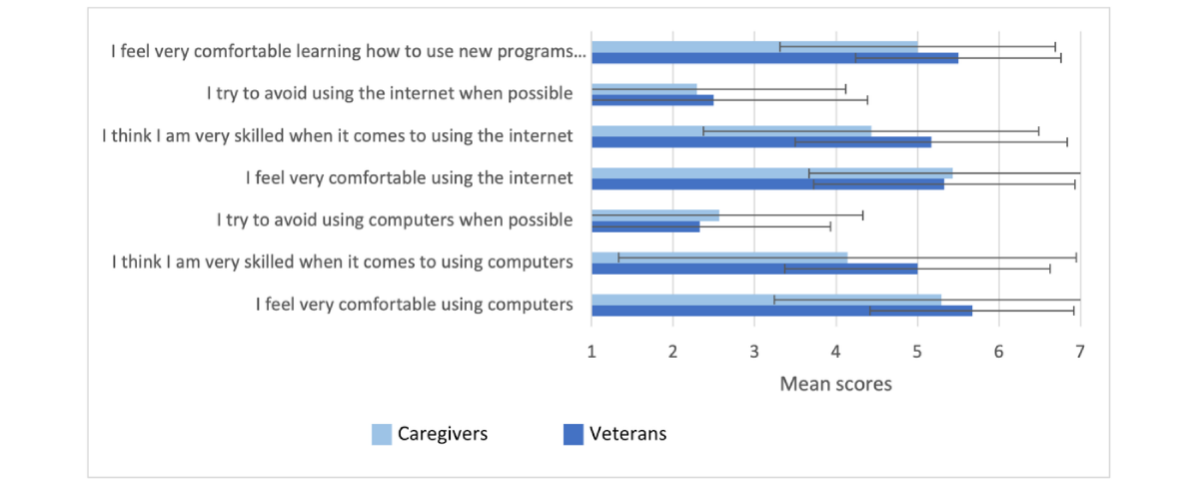
Comfort with technology.

Most participants (12/16, 75%) were frequent users of technology and used computers for several tasks listed in Table 2. Comfort with using computers and the internet is shown in Figure 4.

**Figure 5:**
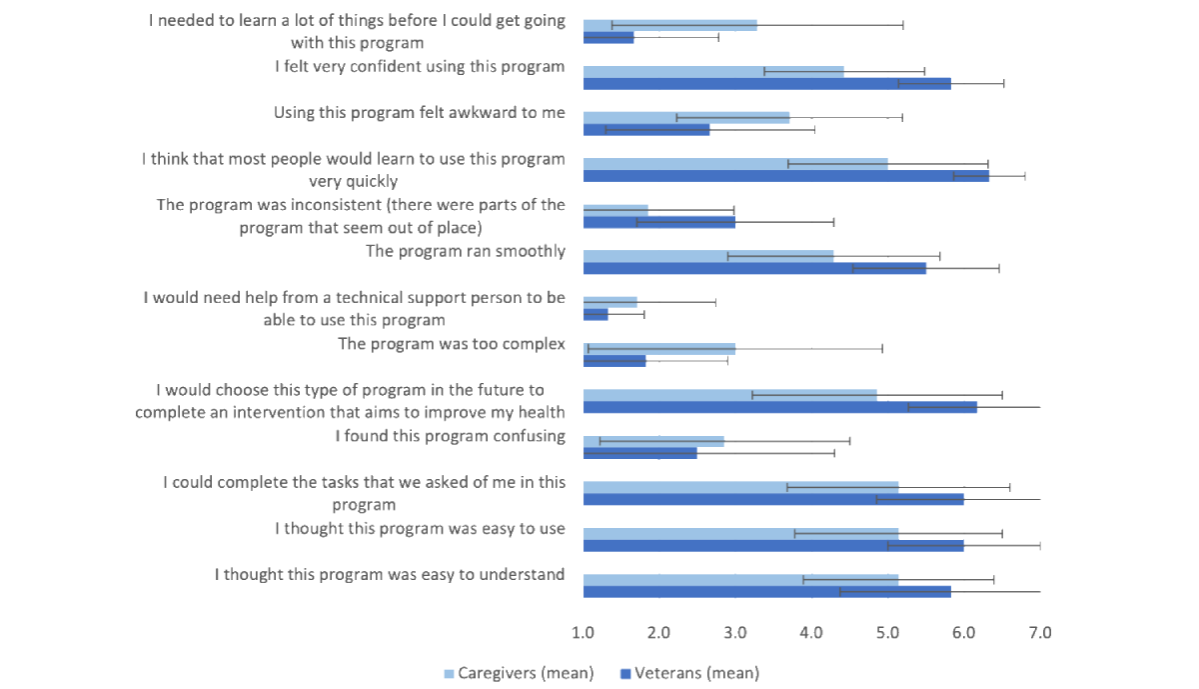
Usability test results.

Participants, especially veterans, rated web-SUCCEED high on usability. Mean scores for each item are provided in Figure 5. Veterans agreed that the site was easy to understand (mean score 5.8, SD 1.5), easy to use (mean score 6, SD 1), and easy to complete (mean score 6, SD 1.2) and were confident in their ability to use it (mean score 5.8, SD 0.7). All veterans (8/8, 100%) noted that they could learn to use the site quickly (mean score 6.3, SD 0.5). Notable negative feedback included “slightly agreeing” that the site was confusing (2/8, 25%) and awkward (1/8, 13%). All veterans (8/8, 100%) agreed that they would choose this type of program in the future to access an intervention that aims to improve their health (mean score 6.2, SD 0.9). Caregivers’ mean scores for each usability item were lower than those of veterans; however, we did not test for statistical differences. Caregivers agreed that the site was easy to understand and easy to use (mean score 5.1, SD 1.4 for both) and easy to complete (mean score 5.1, SD 1.5) and that most people would learn to use the site quickly (mean score 5, SD 1.3). Unlike the veterans, caregivers were neutral in their confidence in using the site (mean score 4.4, SD 1.1). Caregivers also gave slightly high mean scores for negative statements such as “I thought this program was too complex” and “Using this program felt awkward to me.”

Overall, 75% (12/16) of the participants disagreed with the statement that “they would need help from a technical support person” to navigate the website. However, many participants required support from the study team. The key issues included helping participants find the web-SUCCEED link in their email, helping with changing their password from the default, and encouraging them to complete the program. Overall, 13% (2/16) of the participants who accessed the program on their iPad noted that the “next” arrow was not visible. The study team manually reset the password for some participants to reduce participant frustration. For additional security, we included a task that would confirm that the user was human. Participants described this step as frustrating and time-consuming.

Feedback from participants included the following comments by veterans:

It was helpful to me.

It was pretty easy, my wife helped me with it; I’m not that good with computers, [but] it wasn’t that hard to use.

I’ve saved the URL and hope to access it in the future as a resource.

Caregivers were also generally positive in their feedback and noted the following:

I thought it was pretty user-friendly.

Overall, a good program.

Veterans noted that they would use an intervention such as this; recommend this intervention to others; and if permitted, continue to use web-SUCCEED beyond their study participation. Participants completed the modules in the order presented. All participants completed modules in multiple sittings. A veteran noted the following:

Sometimes it felt too long, but you could take breaks.

Many caregivers noted that they were able to navigate with ease, and those who initially found it challenging were able to complete web-SUCCEED with assistance from a member of the study team.

**Table 1:** Demographics of participants involved in usability testing.

Characteristics		Veterans (n=8)	Caregivers (n=8)
Age (years), mean (SD)		66 (18)	58 (16)
Gender (women), n (%)		0 (0)	7 (88)
Race (White), n (%)		5 (63)	5 (3)
Ethnicity (Hispanic), n (%)		1 (13)	1 (13)
Married or in a romantic partnership, n (%)		5 (63)	6 (75)
**Highest grade completed, n (%)**			
	High school or GED^a^	1 (13)	1 (13)
	Some college or 2-year degree	2 (25)	5 (63)
	4-year college	1 (13)	1 (13)
	Higher than 4-year college	2 (25)	0 (0)
**Employment status, n (%)**			
	Employed at a job for pay—full time	2 (25)	1 (13)
	Homemaker—not currently working for pay	0 (0)	1 (13)
	Not employed—retired	2 (25)	2 (25)
	Not employed—disabled	2 (25)	3 (38)
**Financial situation, n (%)**			
	After paying bills, has enough for special expenses	3 (38)	2 (25)
	Has enough to pay bills but little for special expenses	2 (25)	4 (50)
	Has enough to pay bills but only while cutting back	1 (13)	1 (13)
**Number of people available to help, n (%)**			
	≥2	5 (63)	4 (50)
	1	1 (13)	1 (13)
	0	0 (0)	2 (25)

^a^GED: General Educational Development.

**Table 2:** Participants’ uses of computers.

Technology use		Veterans (n=8), n (%)	Caregivers (n=8), n (%)
**Uses the computer...**			
	Everyday	4 (50)	2 (25)
	1-5 times a week	1 (13)	5 (63)
	Less than once a month	1 (13)	0 (0)
**Uses computers for...**			
	Word processing	4 (50)	4 (50)
	Spreadsheets	2 (25)	1 (13)
	Photos	3 (38)	4 (50)
	Games	3 (38)	3 (38)
	Searching for information	5 (63)	5 (63)
	Buying products	3 (38)	4 (50)
	Social networking	2 (25)	3 (38)
	Email	5 (63)	5 (63)
	Searching for health information	5 (63)	3 (38)
	Buying medications or medical supplies	2 (25)	3 (38)
	Communicating with their health provider	4 (50)	5 (63)
	Assisting with making treatment decisions	1 (13)	0 (0)
	Social networking regarding health issues	1 (13)	1 (13)
	Other	1 (13)	5 (63)

### Estimated Cost

The software development phase cost US $100,000 in direct costs paid to the team based in Washington, District of Columbia, responsible for development. This included a project manager and a web designer (ideation and prototyping: US $25,000; building the website: US $75,000). The other substantial contributor to cost was personnel. Project staff (excluding PI time) involved 0.5 full-time equivalent employee masters-level staff during the software development phase who served as the overall project coordinator and helped design the content and guides and a total of 2.25 full-time equivalent employee for the usability testing. All personnel were part time contributors on this project, which may have increased the time needed to conduct the project. Many were in training and were not provided a salary or were compensated via a stipend for their time. Having dedicated, paid personnel for this project from a common source of funding may reduce both the cost and time required to adapt existing programs into a web-based format.

## Discussion

### Principal Findings

Our first goal in this project was to develop and conduct usability testing of a web-based dyadic self-management program for adults managing chronic health conditions. Our second goal was to document the process of doing so to aid future adaptations. There were several key findings. First, we successfully used a systematic and rigorous process that included a multidisciplinary team, multiple stakeholder involvement, and human-centered design principles to adapt a facilitated dyadic self-management support program into a version that was web based and self-guided. Second, we were able to demonstrate the usability of this adapted web-based program for both veterans and caregivers. Third, we found that despite being self-guided, our program required engagement of the study team to encourage completion and solve technical questions.

One of the most important lessons learned from this process was that even “completely self-guided programs” require live technical support and encouragement from study staff. Although it is possible that the pandemic exacerbated usability problems, a recent review by Shaffer et al [17] concluded that the most efficacious programs had study staff maintain engagement with study participants. Other reviews have also found that web-based stress management interventions were most effective when web-based coaching was also provided [39]. These contacts can be useful to provide additional behavior change coaching, encourage active participation, and troubleshoot any challenges or misunderstandings that may arise among users. On the basis of the results of our pilot study and this review, our clinical trial will be using a “flipped classroom” format that uses both the web-based modules and facilitated telephone-based sessions to review the content, keep participants engaged, and solve technical or computer programming–related challenges.

Dyadic health behavior change is an emerging field, with previous studies examining technology-based interventions for patients and their caregivers managing various chronic diseases including cancer and diabetes [21]. One of the most well-known dyadic interventions was the Family Involvement, Optimistic Attitude, Coping Effectiveness, Uncertainty Reduction, Symptom Management (FOCUS) psychoeducational program, which aimed to improve outcomes in family involvement, patient and caregiver optimism, coping, uncertainty management, and symptom management for survivors of breast cancer and their family members. Dyads in the FOCUS program showed improvements in quality of life, emotional and functional well-being, and self-efficacy [22]. Trials of the dyadic CarePartner self-management interventions have also shown that providing self-management support to patient-caregiver dyads improves outcomes when compared with both usual care and patient-only interventions [5,40,41]. Web-SUCCEED builds on the success of these programs by explicitly supporting the role of interpersonal caregiving relationships in the self-management processes. Our newly funded randomized clinical trial of web-SUCCEED will further advance our knowledge of dyadic self-management across different chronic conditions.

The costs of developing multicomponent behavioral programs such as web-SUCCEED are not often published despite it being an important consideration, especially among early career investigators. We found that most of the costs were attributed to software development, followed by study personnel. Resourcing program adaptation through multiple funding sources caused delays and likely introduced inefficiencies in the development process. Future studies should use the cost guidelines that we provide and apply for sufficient funding to conduct both the adaptation and usability testing processes.

We missed the opportunity to ask participants why they did not use the discussion board. There may be several explanations, such as participants’ privacy concerns [42] and insufficient number of participants at any given time to have robust synchronous conversations. Our study team did not provide specific prompts to encourage participation, and participants could have merely forgotten about this feature. This is supported by the findings that participants needed prompts to progress through the program. The literature about the use of study-specific discussion boards is mixed, and their utility remains as an open question. Although some studies have found study-specific social media to support robust engagement, other studies highlight the benefits of using existing social media platforms, including their ubiquitous nature; minimal skills training of study participants; and vibrant, user-friendly graphics [43]. We also noted that, unlike the Veteran and Family Council, which recommended discussion boards to develop a sense of community, focus groups stated a preference for connecting live, either in person or over the telephone. As web-based gatherings have become normalized since the COVID-19 pandemic, future studies should consider this strategy of fostering a sense of community within the study context and making this forum available to both current participants and alumni.

Participants’ time to complete the program was considerably longer than expected. Although we had expected to deliver the program within 6 to 8 weeks, participants required 12 to 16 weeks. In most cases, delays were caused by participants forgetting about their study progress and participation. This was exacerbated by the fact that only the web design company had access to information about the participants’ progress. Although this additional step was a safeguard, it introduced substantial inefficiencies. Assigning a study team member to have administrative access allowed us to track progress, and we discovered that some participants viewed the introductory video but not the modules. Refining study procedures to track progress, engaging participants through weekly reminders, and offering technical help improved recruitment and retention.

On the basis of this experience, we offer the following recommendations for future efforts to adapt dyadic, facilitated behavioral interventions into self-guided, web-based versions (Textbox 1).

A strong multidisciplinary team with the required content, methodological, and technical expertise is critical. We recommend that research teams secure concurrent funding to support adaptation and usability testing to ensure quick iterations and more efficient use of resources. Interventions should follow the same conceptual framework as the original program while incorporating a usability framework. Research teams should follow a rigorous and iterative process that involves ideation, prototyping, refining, and usability testing, as is demonstrated in this study. An underreported domain in technology development is the cost of developing technology-enabled programs. Research teams should develop cost measures that include not only the cost of initial development but also the cost of maintenance and hosting. On the basis of our experience and current literature, study-specific discussion boards are controversial. If used, research teams should proactively engage users through both reminders and by initiating discussions. Tracking user engagement and tracking the success of each engagement strategy can provide valuable lessons about the usability and eventual scalability of programs. This can be accomplished by training a core research team to have administrator-level access to the software analytics. As with any study, research teams should develop protocols to maintain contact with participants and track the success of each strategy; use validated measures, including for usability; and finally, elicit feedback from participants via semistructured interviews.

**Recommendations.**

- Assemble a multidisciplinary team with content, methodological, and technical expertise.
- Secure concurrent funding to support all steps of the adaptation and usability testing.
- Maintain integrity to the theoretical underpinnings of the original program.
- Use a strong usability framework to guide the adaptation.
- Follow an iterative process involving ideation, prototyping, refining, and usability testing.
- Develop and track cost, including cost of maintenance and hosting.
- Proactively engage participants in program-specific discussion boards.
- Develop and track the success of engagement protocols.
- Train the study staff to use software analytics to track user engagement in real time.
- Use validated measures to measure usability and acceptability.
- Elicit feedback about usability and acceptability of interventions from participants via semistructured interviews.

### Strengths and Limitations

A key strength of the study was including extensive stakeholder engagement and feedback from a Veteran and Family Advisory Board; a multidisciplinary team of content experts, methodologists, administrative personnel, web designers, and software engineers; and study participants. Furthermore, we used a rigorous and iterative approach that allowed us to continually improve our product from an initial telephone-based program to a prototype of web-SUCCEED to a beta version of web-SUCCEED that we tested for usability. Our study also had important limitations in addition to those noted previously. First, the logistical challenges of having individuals attend multiple in-person sessions was a key barrier to our ability to conduct think-aloud sessions. We sought to pivot to conducting these over the telephone, but participants were less willing to engage in this way. This step could have given us important data regarding the subjective experience of navigating web-SUCCEED. Second, focus groups comprised only veterans. Caregivers’ input may have modified the web design in ways that were different from the veterans’ input. This may be a reason why caregivers’ usability scores were generally lower than those of the veterans. However, it should be noted that caregivers provided feedback about the look and feel of the inanimate wireframes and were otherwise involved in all aspects of developing SUCCEED and usability testing. Third, owing to funding gaps, the adaptation and usability testing process of web-SUCCEED took longer than anticipated. To avoid this, researchers should consider applying for funding to conduct all aspects of adaptation that are described in this paper. We are currently launching a randomized clinical trial to evaluate the effectiveness of web-SUCCEED and have enhanced our procedures to address the limitations that we described in this paper, including improved staffing, regularly scheduled calls with participants, procedures to track engagement, and modifications to the software to improve usability.

### Conclusions

The COVID-19 pandemic has been a catalyst to rapidly develop and deploy tools that enhance technology-enabled care [44]. The pandemic has also spurred new frameworks that can be used to adapt existing interventions to different contexts [45,46]. Although our study predates the pandemic, the principles we followed are consistent with the new frameworks. Adapting existing interventions is potentially cheaper than creating programs de novo, as the empirical base of the programs has been established. Our experience of developing web-SUCCEED and recommendations provide a road map to adapt other dyadic self-management programs. In parallel, more studies are needed to expand the body of knowledge about adapting and testing traditional-modality interventions into web-based interventions and to understand the strengths and limitations of each approach.

## Abbreviations

FOCUS: Family Involvement, Optimistic Attitude, Coping Effectiveness, Uncertainty Reduction, Symptom Management
PI: principal investigator
SUCCEED: Self-care Using Collaborative Coping Enhancement in Diseases
VA: Department of Veterans Affairs
VAPAHCS: Veterans Affairs Palo Alto Health Care System

## Data Availability

The data sets generated and analyzed during this study are available from the corresponding author upon reasonable request and consistent with our authorization from our institutional review board.
